# The Action of Phosphorus as a Stimulant

**Published:** 1875-02-15

**Authors:** John Brunton


### THE ACTION OF PHOSPHORUS AS A STIMULANT.

By John Brunton, M.A., M.D.

*From the London Lancet, January*, 1875.

NEARLY, if not all the writers upon the therapeutic action of phosphorus are agreed in this, that in certain conditions of extreme physical depression or exhaustion, the action of this drug, in full doses, is of exceeding value. Patients who to all appearance are sinking rapidly, are stimulated and tided over their difficulty. In those eruptive fevers which have a definite term, and towards the close of whose course, just before the turn, the exhaustive depression is very great, phosphorus in stimulating doses acts like a charm.

The difficulties that have heretofore been met with in the administration of this drug, as regards stability, solution, absorption, and taste, and the consequent uncertainty accruing thereto, have thrown it into desuetude; but now that convenient and certain formulæ exist, a fair opportunity has arisen for testing the marvelous powers of this agent. On this account I take an early opportunity of testifying to its powers.*

Five weeks ago, Mrs. M——, aged forty-seven, was in the twelfth day of typhus fever. Her depression was very great. She lay in bed cold, nearly pulseless, semi-unconscious, and with difficulty aroused, quite unable to move in bed; in short, she appeared moribund. One-twelfth of a grain of phosphorus was administered every two hours, with marvelous effect. The revivifying power was very marked, by return of heat, pulse, consciousness, and general power. Next day she had the turn, and has recovered admirably.

J. C——, aged forty, a railway goods guard, from Peterborough, came to me on the 12th August, suffering from diarrhœa, which at that time and season was very prevalent. In a day or two he had the appearance of having something more than mere diarrhœa. He became too ill to get out, and took to his bed. On examination, I found the characteristic rose-colored spots of typhoid fever, and he had all the other symptoms present. His fever continued gradually to increase till the 29th August, when he said to me, "I am going to die"; and he looked like it. His conjunctivæ were injected, his breath cold, his skin cold and clammy; pulse 48, very weak and compressible; voice whispering; temperature 96.4°. He was in a condition of extreme depression. I at once administered phosphorus, in doses of one-twelfth of a grain, every two hours, and I was surprised to find on my next visit, eighteen hours after, when he had taken three-quarters of a grain of the drug, that he had quite revived. His skin was comfortably warm, eyes not so suffused, voice more natural; pulse 72; temperature 99°. I immediately stopped the phosphorus and gave nitric acid. Since that he has gone on prosperously, and is now convalescent.

Of course many more cases are needed to form a good induction, but I record these two for the purpose of inducing others to try the remedy. Heroic doses need careful watching, and I am sure the formula appended is most stable and active, and it is not unpalatable. I do not think that I should care to go beyond one, or at most two grains of the drug, divided over two days. The second case is a good illustration that the drug is to be suspended when the desired effect is produced.

FORMULA.

| | |
|---|---|
| Etherial tinct. phosp. (gr. $\frac{1}{3}$ to ʒ i.), | ʒ iii. |
| Spt. vin. rect., | ℥ ss. |
| Glycerin. anhydr., | ad ℥ iss. |

One teaspoonful as a dose.

* For full information on the subject, the reader will do well to refer to the recently published work, "Free Phosphorus in Medicine," by J. Ashburton Thompson, pp. 32, 33 and 165.

FUNCTIONS OF THE SPLEEN.—M.M. Picard and Malassey communicated to the *Soc de Biologie* at its session Nov. 7 (reported in *Gaz. Med. de Paris*) the results of some experiments as to the function of the spleen, very similar in many respects to those of Tarchanoff, to which they make no reference. Their experiments were, however, conducted with reference to the red and not the white globules of the blood apparently, and the condition of the blood in this respect was very exactly noted.

They experimented on dogs, and cut the nerves of the spleen, and observed the effect of excitation of the same on the condition of the blood, as to the number of globules and the capacity for absorbing oxygen. These particulars were also noted before the section of the nerves, and before and after the division of the sympathetic. The following is the summary they give of the results:

1. The paralysis of the nerves of the spleen is followed by an augmentation of the globular richness of the blood and its richness in hæmoglobine.

2. During the excitation, on the other hand, a diminution of the same is produced.

3. We observe the inverse phenomena in other regions which we have studied in regard to these points.

The investigation is to be carried on by still further series of experiments.

---

BROWBEATING HYSTERIA.—A correspondent of the *Boston Medical and Surgical Journal* gives the following account of the treatment of a typical case of hysteria by Dr. Weir Mitchell: Patient was a young lady who came to the doctor from Rhode Island for treatment. She had been in bed for months. The medical experience of Rhode Island had been exhausted. Dr. W. A. Hammond advised a longer continuance in bed. Dr. Mitchell made three visits ere he began treatment. The peculiarities of the case were spinal weakness and an inability to straighten the lower extremities. At his fourth visit the doctor requested his patient to straighten her limbs. "But I can't." "But you *can*. Are they never straightened at night?" "Yes, doctor. No one ever asked me that question." The legs were straightened with but little difficulty. "Now, be kind enough to sit up." "But that is impossible; I have not been able to do it for two years." "You are able now. Please sit up." Patient sat up. "Bring her wrapper, hose and slippers, and put them on; put on a necktie; belt her waist. Now I wish you to stand." The patient now began to cry. "Good morning," said the doctor, taking his hat. "Where are you going, doctor?" "I am going away. I never attend patients who do not obey me." "Come back, doctor; I will obey you." "Then please stand up." She stood up. "But, doctor, it makes me so dizzy." "I expected it. Take my arm." She took his arm. He led her slowly out of the room, down stairs, and out of doors. She returned without aid and *did not go to bed again*. She was cured. This is given as a sample of the doctor's treatment of hysteria. He is never unkind, never rough, but inflexible, quick in manner, decided in speech, yet gentle and exceedingly polite.—*Detroit Review.*

---

RELATION OF OZONE TO DISEASE.—Dr. J. F. Baldwin (*Amer. Jour. Med. Sciences*, Oct., 1874) gives us an interesting collection of facts on this topic. These facts, he thinks, teach that *ozone influences the general health only in so far as it purifies the air by destroying*—not the living germs of disease, but—*the products of decomposition.*

---

HYSTERIA AND SPINAL IRRITATION TREATED BY CENTRAL GALVANISM.—Dr. Hutchinson (*Archives Electrology and Neurology*, May, 1874,) details three cases in which the happiest results followed the passage of electrical currents through the entire spinal cord.

THE PATHOLOGY OF PURPURA HÆMORRHAGICA.—With the extension of our knowledge of the intimate pathology of disease, we may hope for a corresponding increase of our power of diagnosis and of rational treatment. Any fresh discrimination of forms of disease which have been confused under one title, owing to the mere fact of having one symptom in common, must therefore be welcomed. Dr. B. W. Richardson, whose researches in this direction have been so fruitful, has made another contribution of the kind in the disease known as purpura hæmorrhagica, to which he has applied his observations on the properties of colloids. In a paper read before the Medical Society on the 9th November, he divides the disease into three classes, distinct in their etiology, diagnosis, and treatment: the *aqueous*, in which the water of the blood is in excess, and the colloids relatively decreased; the *saline*, where some saline substance holds the colloidal fibrin in undue solution; and the *vascular*, where there is a degenerative change in the capillaries, and rupture or transudation is facilitated. Dr. Richardson believes that the character of the eruption varies in these several forms.—*London Lancet.*

MEDICINE IN JAPAN.—The Island of Yesso is becoming more and more prosperous. An American physician has founded as many as five hospitals for the natives. He has established a regular clinique, and gives lectures to the students. These lectures are published, with illustrations, in a monthly periodical written in the Japanese language.—*Ibid.*

